# Identity and motivation of adolescent student-athletes in school and elite sport: an investigation of the relationship within- and cross-domains

**DOI:** 10.3389/fspor.2025.1625068

**Published:** 2025-07-22

**Authors:** Maike Niehues, Lara Wille-Rex, Jeffrey Sallen

**Affiliations:** ^1^ Institute of Human Movement Science, University of Hamburg, Hamburg, Germany; ^2^ Educational Sciences, Faculty of Human Sciences, University of Potsdam, Potsdam, Germany; ^3^ Faculty of Sport Science, Leipzig University, Leipzig, Germany

**Keywords:** dual career, role identification, competitive sport, linear regression, secondary school

## Abstract

**Introduction:**

Previous studies have highlighted the effects of motivation and identity on dual careers (DC) separately.

**Methods:**

The present study investigated the mutual relationship among 495 student-athletes (mean age = 17.0, males = 56.6%) in upper secondary school. The Student Identity Measurement Scale (SIMS), Academic Identity Measurement Scale (AIMS), and Student-Athletes’ Academic and Athletic Motivation Scale (SAAMS) were used in a cross-sectional design.

**Results:**

Findings revealed that student-athletes expressed higher identity than motivation within each domain. Athletic identification and motivation were stronger than those for academics. Correlation models revealed strong relations between identity and motivation within a domain, but not across domains. Gender differences emerged, with female student-athletes reporting higher academic identity and motivation compared to males.

**Discussion:**

The study supports the notion that balancing both academia and sports is vital to prevent identity foreclosure and enhance a successful DC. Additionally, the need for individualised support and talent identification strategies for student-athletes is emphasised.

## 1 Introduction

In various fields requiring exceptional abilities, such as athletics, individuals engage in competitions to showcase their skills. Alongside pursuing a career in sports, athletes often juggle academic endeavours, a phenomenon known as dual career [DC; (1)]. Student-athletes, as they are commonly referred to, face the challenge of balancing both domains to avoid prioritising one over the other (2). To avoid a prioritisation of one career, student-athletes need to be supported (3–6).

Recognising the complexities of this balancing act, Wylleman and Rosier (7) developed the Holistic Athletic Career model (HAC) to illustrate the transitions faced by student-athletes at psychological, psycho-social, and economic levels. The HAC model underscores the need for student-athletes to allocate time and energy across various life domains necessitating fluid transitions (8). This constant oscillation between academic and athletic responsibilities can lead to a blurred sense of self-identity among student-athletes. Woodruff and Schallert (9) refer to this sense of self as a motivational sense of self indicating that student-athletes' identity and motivation are inseparable. Understanding the interconnectedness of motivation and identity is crucial for fostering successful DCs, though existing research on their effects remains inconclusive (10, 11). Despite the complexities involved, Stambulova and Wylleman (12) advocate for further exploration of motivational and identity aspects to facilitate successful DCs.

## 2 Theoretical background

### 2.1 Student-Athletes' identities in DCs

The acquisition of an identity presupposes that individuals' identities are shaped by social attributions and personal adoption forming a self-concept derived from a unique combination of social identities (13). In identity theory, role identification and identity are used as terms to describe “one aspect of an individual's wider self-concept, encapsulating an individuals's subjective assessment of who they are, and how they fit with their social world in relation to others” (14). The present study will use these terms synonymously. Regarding student-athletes' identity, these are primarily formed in the athletic and academic domain. In this sense, Snyder (15) has categorised student-athletes into four types based on their levels of commitment to academic and athletic ranging from scholar-athlete to pure athlete. Previous research has employed qualitative and quantitative methods to investigate student-athletes' identity (13). A dominant measurement instrument used to gauge the intensity of athletic identification is the Athletic Identity Measurement Scale (16–18). The participants in these studies comprised diverse groups from different cultural backgrounds (Ireland, Germany, USA), with varying educational levels (school, higher education) and age ranges from 12 to 58 years. Notably, student-athletes primarily identify themselves as athletes as evidenced by stronger athletic identification among elite-level competitors (16, 19–22). A strong athletic identity is especially pronounced during adolescence when compared to later stages of athletic development as highlighted in a systematic review of youth athletes (23). This strong athletic identity can potentially detract from academic pursuits highlighted by a negative relationship between athletic identity and academic mastery as well as performance goals (24). Moreover, Watson et al. (25) explored NCAA Division-I student-athletes' role separation revealing that high athletic identity was associated with a low degree of role separation which positively impacts their well-being. Gender differences also come into play with US-American female student-athletes generally exhibiting stronger academic identities than their male counterparts (18). Moreover, the evolution of identification over time reveals a shift from athletic to academic priorities as student-athletes mature (26) potentially leading to conflicts that impede successful DC completion. These findings highlight that a role conflict can occur when the demands of one identity interferes with meeting the demands of the other identity (14). As a result, the role interference can threaten the completion of a successful DC and might lead to athletic identity foreclosure as student-athletes do not explore occupational or ideological alternatives (27). In other words, while a strong athletic identity can underpin resilience and motivation, fostering a well-rounded, multifaceted sense of self, it is equally important to ensure a balanced pursuit of both athletic and academic aspirations to support the development of a comprehensive and adaptable identity.

### 2.2 Student-Athletes' motivation in DCs

Besides identity, academic and athletic motivation can impact successful DC pathways as motivation serves as a driving force for individuals' efforts and aspirations toward achieving desired outcomes (28). Research on DC motivation revealed a variety of approaches (29, 30) with two successful methods for measuring DC motivation simultaneously among student-athletes in higher education (31) and in secondary school (32). Studies have found a significant interplay between mental health and motivation among student-athletes active in Gaelic games (33) crucial for successful DC pursuits. Gender disparities manifest in differential DC motivation levels with males expressing higher athletic motivation and females exhibiting higher academic motivation (34–37). Similarly, elite-level competitors display heightened athletic motivation (36, 38). These studies were conducted across different cultural contexts, including various European countries and the USA, focusing on similar age groups (18–29 years) and involving participants with comparable educational backgrounds (higher education institutions). As a result, athletic motivation and the desire to pursue a professional athletic career detracts from academic success (31). Considering previous findings from a positive perspective, a strong motivation towards athletic achievement and the aspiration to pursue a professional athletic career can complement academic pursuits when balanced effectively.

### 2.3 Relationship of DC motivation and identity

Generally, DC identity and motivation are strongly related as student-athletes who identify with their academic and athletic role simultaneously will likely be motivated for a DC. This strong relation is highlighted by a meta-analysis which revealed a positive association of academic identity and motivation with academic achievement (39). Eccles [(40), p. 81] highlighted the relationship of motivation and identity by pointing out “that the motivational aspects of identity and identity formation processes […] are directly related to [the] socio-cultural expectancy-value model of motivated behavioral choices”. Studies exploring DC motivation and identity concurrently (41–43) revealed their mutual influences. For example, academic and sport contexts influence identity and motivation considerably among Portuguese and Brazilian student-athletes (41). Woodruff and Schallert (9) explored DC motivation and identity in more depth by interviewing nine NCAA Division I student-athletes in a grounded-theory approach. The researchers highlighted that student-athletes' experiences have first an effect on their motivation. Changes in student-athletes' motivation, in turn, affect their identity. In contrast, Oyserman (44) summarises findings from various studies regarding her proposed identity-based motivation theory. Findings suggest that motivations are shaped by one's identity and influenced by contextual factors with individuals gravitating towards actions that align with their identities. While Woodruff and Schallert (9) as well as Oyserman (44) have elucidated effects of motivation on identity and vice versa, further research exploring the mutual relationship between motivation and identity is warranted, particularly across domains. To date, only one study has examined the relationship across the athletic and academic domain among NCAA Division III student-athletes. Love and Rufer (45) reported that academic identity was positively correlated with both athletic identity and academic motivation. Furthermore, athletic identity was found to be positively associated with athletic motivation, while exhibiting a negative association with academic motivation. Conversely, academic motivation was negatively correlated with athletic motivation.

### 2.4 Study objectives

Given the predominant focus in existing research on the impact of identity or motivation on a DC, this study aims to fill a critical gap by investigating the mutual relationship between identity and motivation. This two-way interaction has not been explored in previous research, making it a central objective of the present study. By elucidating the non-directional relationship between DC motivation and identity, the present study aims to advance understanding in this area. Specifically, the study seeks to address the following research questions:

1. To what extent do student-athletes’ differ in their expression of motivation and identity
  (a) within a domain (academic identity vs. motivation and athletic identity vs. motivation), as well as
  (b) across the two domains (academic vs. athletic identity and academic vs. athletic motivation)?
2. To what extent does student-athletes’ academic and athletic identity relate to their academic and athletic motivation?
3. To what extent do male and female student-athletes differ with regards to their expression of academic and athletic identity as well as motivation?

## 3 Materials and methods

### 3.1 Study design and procedure

The study employed a cross-sectional design to investigate the mutual relationship between student-athletes' identity and motivation. Data collection involved administering a self-disclosure online questionnaire to student-athletes within their schools with trained staff members' guidance and supervision. Participation included three Austrian schools in one Austrian federal state and nine German schools spanning five German federal states. Data collection occurred between March and July 2022. All research procedures adhered to the principles outlined in the 1964 Helsinki declaration and its subsequent amendments or comparable ethical standards.

### 3.2 Instruments

The online questionnaire encompassed socio-economic questions, including questions on student-athletes' sex, age, elite sport, and squad level. To assess the student-athletes' *athletic identity*, the German version of the Athletic Identity Measurement Scale by Schmid and Seiler (17), adapted from Brewer et al.'s (1993) original scale, was employed (McDonald's *ω* = .89). A sample item is: “Sport is the most important part of my life”. For the investigation of *academic identity*, a parallel instrument, the Student Identity Measurement Scale developed by Engström (46), was utilised and adapted for the German context (McDonald's *ω* = .80). Sample items include statements like “I consider myself a student”. Both identity measurement instruments consisted of ten items each, and responses were provided on a six-point Likert scale ranging from 1 (strongly disagree) to 6 (fully agree). To gauge *athletic and academic motivation*, the Student-Athletes' Academic and Athletic Motivation Scale (SAAMS) created by Niehues et al. (32) was used. The SAAMS comprised 18 items each for academic and athletic motivation, rated on a 5-point Likert scale from 1 (strongly disagree) to 5 (fully agree). An example item from the SAAMS is: “Competencies that I acquire in school/sport are helpful to me”. Missing data were addressed through listwise deletion.

### 3.3 Sample

A total of 495 adolescent student-athletes (56.6% male, 43.0% female, 0.4% non-binary) participated in the study. All participants met the predefined criteria for being an athlete in (pre-)elite sport participation, as outlined by Araújo and Scharhag (47). These criteria include: (1) engaging in dedicated training aimed at enhancing athletic performance, (2) regularly competing in sports events, (3) holding official athlete registrations with a national sport federation, and (4) pursuing sports training and competitions driven by personal interest. Furthermore, all participants attended the secondary level of an elite sport school with DC assistance programmes (48), which provides additional support for adolescent student-athletes in specialised training environments. The majority of the participants belong to the elite level within their respective sports and age groups, as evidenced by their membership in a squad (see Table 1). Nonetheless, most student-athletes are still in the adolescent development stage, and have not yet reached senior-level competition (i.e., they are primarily at the pre-elite level). Additional information regarding the sample characteristics can be found in Table 1.

**Table 1:** Detailed sample description of student-athletes.

	Total sample	Females	Males
	(*N* = 495)	(*N* = 213)	(*N* = 280)
Age (years)	*M* (SD)	*M* (SD)	*M* (SD)
	17.0 (1.1)	16.9 (1.1)	17.0 (1.0)
Elite squad level	*n* (%)	*n* (%)	*n* (%)
A & B squad (international level)	30 (6.1)	12 (5.6)	18 (6.4)
C squad (national level)	99 (20.0)	49 (23.0)	50 (17.9)
D/C squad (regional level)	61 (12.3)	23 (10.8)	36 (12.9)
D squad (regional level)	126 (25.5)	50 (23.5)	76 (27.1)
other squad level	45 (9.1)	21 (9.9)	24 (8.6)
no squad level	134 (27.1)	58 (27.2)	76 (27.1)
Groups of Olympic sport disciplines	*n* (%)	*n* (%)	*n* (%)
Endurance sports^a^	80 (16.2)	40 (18.8)	40 (14.3)
Team sports/sports games^b^	246 (49.7)	95 (44.6)	150 (53.6)
Strength and speed-strength sports^c^	66 (13.3)	32 (15.0)	34 (12.1)
Combat sports^d^	31 (6.3)	14 (6.6)	17 (6.1)
Artistic composition sports^e^	14 (2.8)	7 (3.3)	6 (2.1)
Multi-discipline sports^f^	35 (7.1)	15 (7.0)	20 (7.1)
Others^g^	23 (5.1)	10 (4.7)	13 (4.7)

^a^ e.g., canoeing, running, rowing, swimming.

^b^ e.g., handball, football, volleyball, water polo.

^c^ e.g., weightlifting, athletics (sprinting, jumping, throwing, shot put).

^d^ e.g., judo, boxing, wrestling.

^e^ i.e., figure skating, cheerleading.

^f^ i.e., triathlon, decathlon, modern pentathlon.

^g^ i.e., equestrian, sport shooting.

### 3.4 Data analysis

The analysis utilised SPSS Statistics (version 28.0, IBM Corp., Armonk, NY, USA) for descriptive data examination and Mplus (version 8.7, Muthén and Muthén, Los Angeles, CA, USA) for multivariate correlation model computations. The motivation and identity scales were z-standardised and the scales' mean scores were calculated for subsequent analyses. The data analysis was structured into three parts.

In the first part, descriptive statistics were computed to address the first research question exploring academic and athletic identity as well as motivation. Paired *t*-tests were conducted for these variables yielding effect sizes (Cohen's *d*) and significance levels (*p*). Specifically, paired *t*-tests were employed to investigate differences in identity and motivation within the academic and athletic domains, as well as differences in identification and motivation across both domains (49).

Addressing the second research question, multivariate correlation models were constructed for the manifest variables academic and athletic identity as well as motivation simultaneously. Pearson correlations (*r*), confidence intervals, and significance values (*p*) were calculated to explore relationships among these variables.

Lastly, gender differences were examined in response to the third research question, excluding non-binary student-athletes due to the small sample size. Unpaired *t*-tests were conducted with academic and athletic identity as well as motivation as dependent variables and sex as the independent variable to assess gender differences. Furthermore, multivariate correlation models were established separately for female and male student-athletes based on their expression of academic and athletic identity as well as motivation as described above. The correlation coefficients of the models were compared following Eid et al. [(50), p. 547f] to identify any statistically significant differences in correlations between the male and female model.

The statistical interpretations were based on Cohen's (51) guidelines. Effect sizes (*d*) were appraised as small (|*d*| = .2), medium (|*d*| = .5), or strong (|*d*| = .8). Correlation strengths were evaluated as weak (*r* ≤ .10), moderate (*r* ≤ .20), or strong (*r* ≤ .50). Confidence intervals were reported at the 95% level with significant differences noted when the interval did not encompass zero. In addition, *p*-values were denoted at *p* ≤ .05, *p* ≤ .01, and *p* ≤ .001 where lower values indicate higher significance levels.

## 4 Results

Addressing the first research question, the results of the descriptive analysis (Table 2) revealed slightly higher mean scores for motivation than identity within the respective domain. The paired *t*-tests (Table 2) corroborated this trend only in the academic domain with a weak effect size. When examining cross-domain differences, the descriptive analysis (Table 2) indicated marginally higher mean scores for the athletic than the academic identity and equal mean scores for athletic and academic motivation. Despite this observation, the *t*-tests (Table 2) did not indicate significant differences within domains.

**Table 2:** Results of the paired *t*-tests for identity and motivation as well as for gender differences using the *z*-standardised values.

Parameter	*M*	*SD*	*M*	*SD*	*t*	*df*	*p*	Cohen's *d*
Expressions in the academic domain								
	Academic identity		Academic motivation					
Student-athletes	−.03	.59	.01	.52	1.70	494	.045	0.08
Expressions in the athletic domain								
	Athletic identity		Athletic motivation					
Student-athletes	.00	.70	.01	.55	0.37	479	.358	0.02
Expression of identity								
	Athletic identity		Academic identity					
Student-athletes	.00	.70	−.03	.59	−0.75	479	.226	0.03
Expression of motivation								
	Athletic motivation		Academic motivation					
Student-athletes	.01	.55	.01	.52	−0.19	494	.425	0.01
Gender differences								
	Female athletes		Male athletes					
Athletic identity	−.01	.64	.01	.74	0.31	476	.380	0.03
Academic identity	.15	.61	−.18	.53	−6.30	491	<.001	0.57
Athletic motivation	−.05	.51	.06	.58	−5.10	491	<.001	0.19
Academic motivation	.14	.49	−.10	.52	2.13	482	.017	0.56

**Figure 1:**
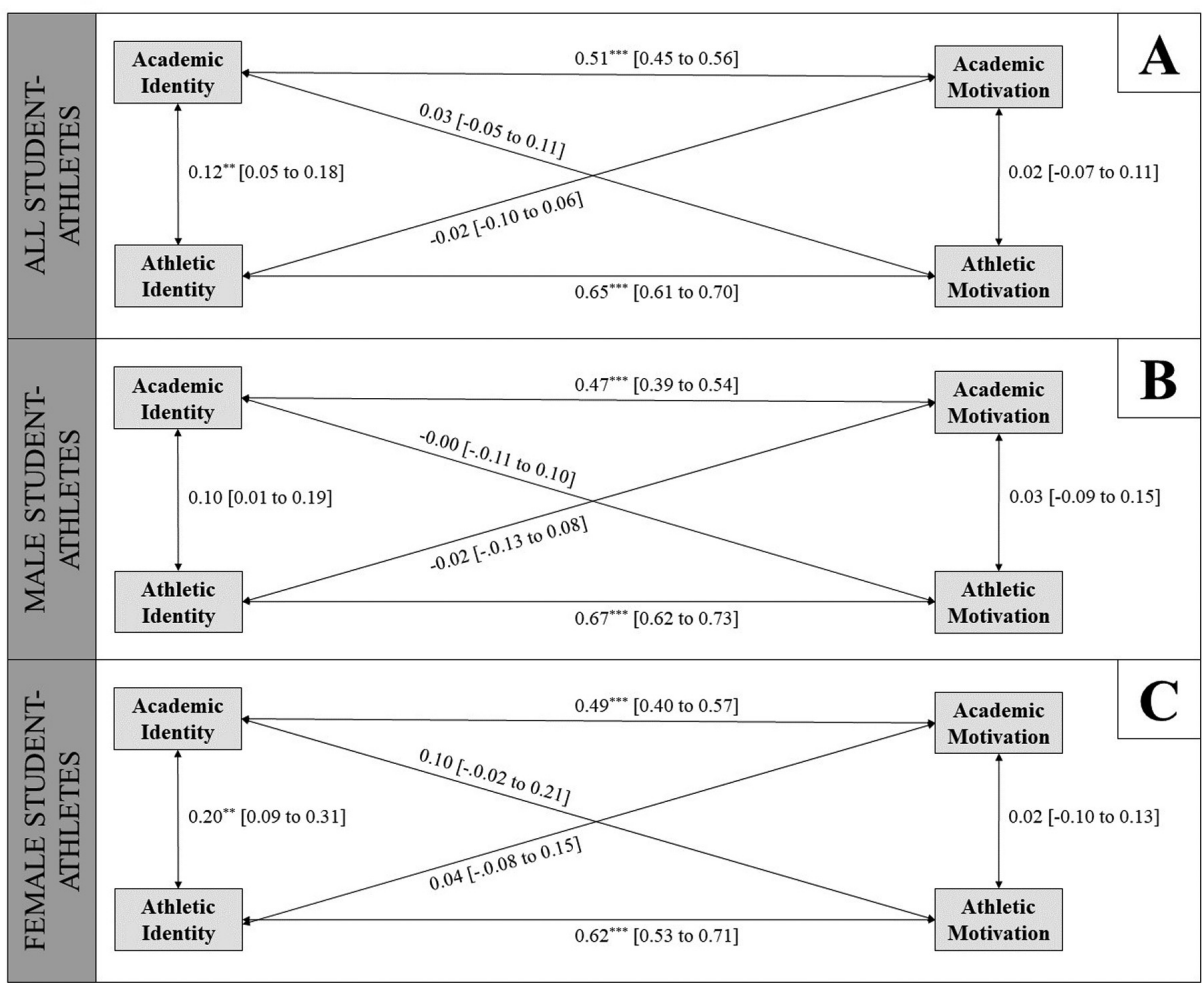
Results of the correlation models for **(A)** all student-athletes (*N* = 480), **(B)** male student-athletes (*N* = 270), and **(C)** female student-athletes (*N* = 207). Standardised correlation coefficients (*r*) and 95% confidence intervals are presented. *p ≤ .05, **p ≤ .01, ***p ≤ .001.

For the second research question, the correlation analysis results (Figure 1) exhibited significant within-domain correlations underscoring the significantly strong relationship between academic motivation and identity as well as between athletic motivation and identity. The only cross-domain significant correlation was found for academic and athletic identity though characterised by a weak correlation. All other within- and cross-domain correlations did not yield significance.

Exploring gender differences as per the third research question, *t*-test outcomes (Table 2) unveiled differences in academic identity with female student-athletes demonstrating higher scores in this domain compared to their male counterparts. Differences were also observed for athletic and academic motivation with females reporting higher academic motivation and males exhibiting elevated athletic motivation. In contrast, no gender differences were detected concerning athletic identity. The comparison of the correlation models (Figure 1) for male and female student-athletes did not differ significantly (Table 3), although male student-athletes displayed no correlation between academic and athletic identity while the female as well as the entire study sample exhibited a weak correlation.

**Table 3:** Results of the comparison regarding the correlation analysis for gender.

Correlation comparison	*z*-value	*p*-value
Academic identity vs. academic Motivation	−0.279	.390
Academic identity vs. athletic motivation	−1.079	.140
Academic identity vs. athletic identity	−1.101	.135
Athletic identity vs. academic motivation	−0.645	.259
Athletic identity vs. athletic motivation	0.922	.178
Athletic motivation vs. academic motivation	0.108	.457

## 5 Discussion

The present study marks a significant advancement as the first to simultaneously investigate student-athletes' identity and motivation in an upper secondary school setting utilising the newly developed SAAMS (32). The findings offer valuable insights into the intricacies of student-athletes' identity and motivation within the academic and athletic domain as well as across both domains.

Regarding the first research question, the results align with those of Love and Rufer (45), who found that academic identity is positively associated with both athletic identity and academic motivation, while athletic identity is also positively linked to academic motivation. Moreover, the results indicated slightly higher levels of motivation compared to identity within each domain. This observation contrasts with the notion proposed by Woodruff and Schallert (9) of the inseparable link between identity and motivation. While prior studies have revealed higher expressions in the athletic than the academic domain (16, 19), such findings were not replicated in the present study. Heightened athletic identification and motivation pose the risk of prioritising an athletic career path (2), potentially resulting in an educational gap post-athletic career termination and posing a risk of identity foreclosure for student-athletes (27). By compromising oneself to the athletic identity solely, student-athletes are in danger of “losing themselves” in the progress. Promoting DC motivation and identity among student-athletes can contribute to their holistic development and academic success (39). The present sample seemingly strikes a balance between their academic and athletic pursuits potentially attributed to the DC support services tailored to them (52, 53).

In relation to the second research question, the results confirmed the close association between motivation and identity. Strengthening DC motivation can play a pivotal in averting identity foreclosure and guiding student-athletes along a successful DC pathway (2). Similarly, a robust DC identity in both spheres can bolster DC motivation, aligning with Oyserman's (44) assertion that motivation is shaped by the identity forged within particular contexts, in this case, the academic and athletic realms.

Lastly, the gender differences highlighted in the third research question underscored that female student-athletes exhibit higher academic identity and motivation than their male counterparts. This disparity mirrors findings from prior research investigating gender differences in DC identity and motivation (18, 34–37). The gender inequities prevalent in elite sports, including unequal pay, benefits, and underrepresentation of women in leadership, may drive females towards prioritising their academic pursuits as a safeguard (54, 55). To promote a more balanced and sustainable DC among female student-athletes, targeted support strategies are essential for females, ensuring that their DC identity and motivation are nurtured concurrently and fostering their overall holistic development rather than prioritising one over the other.

The study's strengths include its initial exploration of the mutual relationship between DC identity and motivation. Moreover, the representative sample of German and Austrian student-athletes in upper secondary school provides reliable findings.

While the study showcases notable strengths, certain limitations warrant consideration. The absence of longitudinal data precluded an examination of the interactive effects of academic and athletic identity on motivation and vice versa. However, Woodruff and Schallert (9) pointed out that changes in motivation affect a person's sense of self whereas Oyserman (44) indicated a prediction of identity by motivation. Future research could delve into predicting motivation through identity and vice versa using longitudinal data building upon the foundational insights garnered in this study. Additionally, as the data are self-reported, results may be subject to potential biases such as social desirability and response biases, which could influence the findings. Moreover, the sample was limited to participants from Germany and Austria, which may affect the generalisability of the results due to regional and cultural differences. Additionally, factors such as participation in DC assistance programmes and the specific sport type may influence the levels of identity and motivation, further limiting the broader applicability of the findings.

## 6 Conclusion

The study provides valuable insights into the complex relationship between identity and motivation among student-athletes shedding light on the expressions of motivation and identity within and across the academic and athletic domains. Findings underscore the importance of maintaining a balance between both domains to navigate a successful DC pathway without prioritising one over the other, thus, minimising the risk of identity foreclosure and educational gaps post-athletic career. Gender differences highlight the disparities in opportunities and outcomes in elite sports emphasising the pivotal of academic pursuits as a potential backup plan for female student-athletes facing unequal prospects in the athletic realm. The study's innovative approach of examining the mutual relationship between identity and motivation in a DC setting contributes to bridging the gap in existing literature and offers a comprehensive understanding of how these factors interact to shape student-athletes' DC trajectories. While the research represents a significant advancement in understanding the intricacies of DC identity and motivation, future studies with longitudinal data could further explore the dynamic interplay between identity and motivation, providing deeper insights into the factors influencing student-athletes’ success in managing DCs. In terms of practical implications, educational and sports organisations should develop psychological interventions within holistic DC assistance programmes and DC development environments and support systems that foster adaptive identity development and enhance motivation. Such interventions can help student-athletes to effectively manage their DCs.

## Data Availability

The datasets presented in this article are not readily available because the dataset is restricted due to sensitive data. Requests to access the datasets should be directed to Maike Niehues, maike.niehues@uni-hamburg.de.
